# Adjuvant Gemcitabine Versus Neoadjuvant/Adjuvant FOLFIRINOX in Resectable Pancreatic Cancer: The Randomized Multicenter Phase II NEPAFOX Trial

**DOI:** 10.1245/s10434-024-15011-7

**Published:** 2024-03-08

**Authors:** Thorsten O. Goetze, Alexander Reichart, Ulli S. Bankstahl, Claudia Pauligk, Maria Loose, Thomas W. Kraus, Moustafa Elshafei, Wolf O. Bechstein, Jörg Trojan, Matthias Behrend, Nils Homann, Marino Venerito, Wolfram Bohle, Michael Varvenne, Claus Bolling, Dirk M. Behringer, Karsten Kratz-Albers, Gabriele M. Siegler, Wael Hozaeel, Salah-Eddin Al-Batran

**Affiliations:** 1 Krankenhaus Nordwest, Institut für Klinisch Onkologische Forschchung IKF, University Cancer Center (UCT) Frankfurt, Frankfurt, Germany; 2 University Cancer Center (UCT) Frankfurt, Goethe Universität, Frankfurt, Germany; 3 Frankfurter Institut für Klinische Krebsforschung IKF am Krankenhaus Nordwest, Frankfurt, Germany; 4 Krankenhaus Nordwest, Allgemein-, Viszeral- und Minimal Invasive Chirurgie, Frankfurt, Germany; 5 Klinik für Allgemein-, Viszeral-, Transplantations- und Thoraxchirurgie, Universitätsklinikum Frankfurt, Frankfurt, Germany; 6 Gastrointestinale Onkologie, Universitätsklinikum Frankfurt, Frankfurt, Germany; 7 Viszeral-, Thorax- und Gefäßchirurgie, DONAUISAR Klinikum Deggendorf, Deggendorf, Germany; 8 Medizinische Klinik II, Klinikum Wolfsburg, Wolfsburg, Germany; 9 Klinik für Gastroenterologie, Hepatologie und Infektiologie, Universitätsklinikum Magdeburg, Magdeburg, Germany; 10 Klinik für Gastroenterologie, Gastroenterologische Onkologie, Klinikum Stuttgart, Stuttgart, Germany; 11 Hepatologie, Infektiologie und Pneumologie, Stuttgart, Germany; 12 Onkologische Schwerpunktpraxis Celle, Celle, Germany; 13 Hämatologie/Onkologie, Agaplesion Markus Krankenhaus, Frankfurt, Germany; 14 Klinik für Hämatologie, Onkologie und Palliativmedizin, Augusta-Kranken-Anstalt Bochum, Bochum, Germany; 15 Gemeinschaftspraxis für Hämatologie und Onkologie Münster, Münster, Germany; 16 Klinikum Nürnberg Nord/Paracelsus Medizinische Privatuniversität, Medizinische Klinik, Hämatologie/Onkologie, Nürnberg, Germany

**Keywords:** Resectable pancreatic cancer, Neoadjuvant, Adjuvant, FOLFIRINOX

## Abstract

**Background:**

Although addition of adjuvant chemotherapy is the current standard, the prognosis of pancreatic cancers still remains poor. The NEPAFOX trial evaluated perioperative treatment with FOLFIRINOX in resectable pancreatic cancer.

**Patients and Methods:**

This multicenter phase II trial randomized patients with resectable or borderline resectable pancreatic cancer without metastases into arm (A,) upfront surgery plus adjuvant gemcitabine, or arm (B,) perioperative FOLFIRINOX. The primary endpoint was overall survival (OS).

**Results:**

Owing to poor accrual, recruitment was prematurely stopped after randomization of 40 of the planned 126 patients (A: 21, B: 19). Overall, approximately three-quarters were classified as primarily resectable (A: 16, B: 15), and the remaining patients were classified as borderline resectable (A: 5, B: 4). Of the 12 evaluable patients, 3 achieved partial response under neoadjuvant FOLFIRINOX. Of the 21 patients in arm A and 19 patients in arm B, 17 and 7 underwent curative surgery, and R0-resection was achieved in 77% and 71%, respectively. Perioperative morbidity occurred in 72% in arm A and 46% in arm B, whereas non-surgical toxicity was comparable in both arms. Median RFS/PFS was almost doubled in arm B (14.1 months) compared with arm A (8.4 months) in the population with surgical resection, whereas median OS was comparable between both arms.

**Conclusions:**

Although the analysis was only descriptive owing to small patient numbers, no safety issues regarding surgical complications were observed in the perioperative FOLFIRINOX arm. Thus, considering the small number of patients, perioperative treatment approach appears feasible and potentially effective in well-selected cohorts of patients. In pancreatic cancer, patient selection before initiation of neoadjuvant therapy appears to be critical.

**Supplementary Information:**

The online version contains supplementary material available at 10.1245/s10434-024-15011-7.

Pancreatic ductal adenocarcinoma (PDAC) is one of the malignant tumors with the highest mortality. The 5-year overall survival for all pancreatic cancers including all stages was approximately 11% in the US population from 2011 to 2017.^1^ Resection with curative intent followed by adjuvant chemotherapy represents the current standard of care. However, more than 80% of all patients with PDAC are not eligible for curative surgery because of the presence of distant metastasis and/or locally advanced disease at the time of diagnosis.

Despite substantial improvements in pancreatic surgery and effective adjuvant treatment options with more powerful chemotherapy (CTX) regimens over the last years, even tumors with an upfront clearly resectable situation still have an unfavorable prognosis. Furthermore, the overall recurrence rate is as high as 85% and most recurrences occur as systemic liver metastasis early in the course, with a median disease-free survival of only 6.7 months.^2^ Thus, there may be a need for early implementation of a systemic therapy component.^3^

Neoadjuvant or perioperative multimodal therapies have significantly improved pathological response as well as overall outcome of resectable gastroesophageal junction and gastric adenocarcinomas.^4,5^ Immediate systemic therapy has been adopted as a neoadjuvant approach in borderline resectable PDAC on the basis of the findings of several randomized controlled trials (RCTs) demonstrating its survival benefit.^6–9^ Nowadays, neoadjuvant approach is accepted in the National Comprehensive Cancer Network guideline in pancreatic borderline resectable cases in which the cancer is not clearly resectable with upfront surgery because the R+ surgery rate is high without upfront therapy. It is reasonable to assume that systemic chemotherapy and/or radiochemotherapy prior to surgery may have a similar impact on the outcome of resectable PDAC.

Neoadjuvant treatment (NAT) strategy also emerged as an option even in resectable PDAC because of its potential benefits, including immediate treatment of undetectable micrometastases. Furthermore, neoadjuvant treatment enables anticancer therapy that is not hampered by postoperative complications, which are the reason nearly half of the patients with PDAC cannot receive adjuvant therapeutic approaches.^10^ However, NAT may also be associated with a potential loss of eligibility for curative resection owing to tumor growth during therapy. Presurgical attrition occurs in approximately 30% of patients with resectable PDAC, suggesting the possibility of selection bias in studies showing the benefits of NAT.^10,11^ Since there are still conflicting results on survival gain compared with upfront surgery, the real effectiveness of NAT in resectable PDAC remains unclear.^12–14^ Recently pooled data from three randomized controlled trials of NAT for resectable PDAC suggest that this approach should be accepted as new standard of care.^15^ Nevertheless, neoadjuvant treatment of PDAC is currently still considered an experimental approach. The current standard of care is surgical resection followed by adjuvant CTX with either gemcitabine or a combination CTX, e.g., gemcitabine and capecitabine.^16^

Overall, the data provided a strong rationale to evaluate the efficacy and safety of neoadjuvant treatment with FOLFIRINOX (FFX) in patients with resectable PDAC.

## Patients and Methods

### Study Design and Participants

This was an investigator-initiated multicenter, randomized, unblinded, controlled phase II trial. All versions of the trial protocol were approved by the Ethik-Kommission der Landesärztekammer Hessen ethics committee and subsequently by the respective ethics committees of each of the participating centers. The trial is registered with ClinicalTrials.gov (no. NCT02172976). The trial was conducted in accordance with the Declaration of Helsinki. All patients provided written informed consent to participate in this trial.

Patients with histologically confirmed adenocarcinoma of the pancreas were included. For histological confirmation, a maximum of three attempts was allowed. The tumor had to be radiological confirmed as locally limited, resectable [primarily resectable or borderline situation according to the National Comprehensive Cancer Network (NCCN) classification criteria] without distant metastases. Further, the main inclusion criteria were age ≤ 75 years; Eastern Cooperative Oncology Group (ECOG) performance status ≤ 1; adequate hematological, hepatic, and renal function parameters; normal cardiac ejection fraction; no prior pancreas resection; and no prior cytostatic chemotherapy.

Patients were centrally randomized in a 1:1 ratio to the standard treatment arm (A) with upfront surgery followed by six cycles adjuvant gemcitabine (1000 mg/m^2^ on days 1, 8, and 15 on a 28-day cycle) or to the experimental arm (B) with perioperative FFX [irinotecan 180 mg/m^2^, oxaliplatin 85 mg/m^2^, 5-fluorouracil (5-FU) 400 mg/m^2^ bolus, 5-FU 2400 mg/m^2^, and sodium folinate 400 mg/m^2^] on day 1 on a 14-day cycle for 4–6 cycles pre- as well as post-surgery. In arm B, filgrastim should be applied if necessary on days 5–7 of every cycle. Therapy was terminated prematurely for unacceptable toxicity, disease progression, death, or upon patient request. In arm B, surgery was scheduled 4–6 weeks after the last dose of neoadjuvant FFX. The surgeon was responsible for the selection of the adequate oncological resection technique. Centers with experienced pancreatic surgery departments (or established collaboration with such departments) were selected for this trial and the surgery reports were centrally reviewed by an experienced surgeon (T.O.G.).

Radiological examinations [chest computed tomography (CT) and abdominal CT or magnetic resonance imaging (MRI)] were performed before the start of the study and in arm B after the third preoperative cycle and before surgery to assess operability.

### Randomization

Randomization was performed by an interactive web response system (IWRS) on the basis of a sequence generated with permuted blocks and stratified by ECOG performance status (0 vs 1) and resectability (primarily clearly resectable versus borderline resectable). Patients were enrolled by authorized individuals who requested randomization using IWRS integrated in the electronic Case Report Forms (eCRF). Actual assignment to trial groups took place on the server of the independent data management providers (Trium Analysis Online, Munich, Germany) by means of a validated SAS program, which underlies strict access control. The randomization system allocated a unique identification number to every patient and sent a message that included the allocation result to the investigator. The study was open label and no masking was required.

### Study Endpoints

The primary endpoint was overall survival (OS), defined as time from randomization until death from any cause. Secondary endpoints included recurrence/progression-free survival (RFS/PFS), defined as time from randomization until disease recurrence/progression under preoperative CTX, until evidence of recurrence (after surgery had been performed), or death from any cause. Secondary endpoints were perioperative morbidity and mortality, R0 resection rate, and feasibility and tolerability of FOLFIRINOX with regard to the use of G-CSF/filgrastim in primary prophylaxis.

### Statistical Analysis

The initial sample size calculation was based on the median survival of 22.1 months for patients treated with adjuvant gemcitabine within the CONCO-001 trial^2^ and the median survival of 18.8 months for patients treated with neoadjuvant gemcitabine.^17^ Patients with borderline resectable tumors, who were also enrolled in this trial, have a worse survival. Therefore, a median survival of 18 months in the standard adjuvant gemcitabine arm was assumed. Neoadjuvant/adjuvant chemotherapy with FFX was expected to improve median survival with a clinically relevant hazard ratio (HR) of 0.68. Taking into account a dropout rate of 15%, an alpha error of 0.15 (one-sided log-rank test), and a power of 80%, a total of 126 patients at a 1:1 randomization were needed. However, recruitment of the trial was stopped after 40 patients owing to poor accrual.

The intention-to-treat (ITT) population was defined as all patients randomized to participate in the study. The curative surgery population was defined as all patients who are randomized and received radical oncological pancreatic surgery. The curative surgery population was defined by the coordinating investigator on the basis of the surgery reports and database entries.

All parameters were evaluated and reported in an explorative or descriptive manner. The Kaplan–Meier method was used to estimate the distribution of time to event variables. Summaries included the number and percentages of events per treatment group, the median, and 25% and 75% quantiles of survival time as well as the proportion of patients without event at certain timepoints. Event-related data were estimated by the product limit method providing the numbers of events and censored cases, median survival time along with its 95% CI, and compared using the log rank test. Patients without any documentation of events, lost to follow-up, or with early dropout were censored at last observation, i.e., the date of last tumor assessment for progression-free survival and last known survival date for overall survival. Incomplete time-to-event observations were handled as censored measurements and missing data were considered as failure for the primary endpoint.

The analyses were carried out using SAS software.

## Results

Between 5 March 2015 and 23 March 2018, 40 patients were randomly assigned to the phase II part of the NEPAFOX trial. Furthermore, 21 patients were assigned to arm A, with direct upfront surgery without preoperative chemotherapy followed by adjuvant gemcitabine CTX (SOC- arm), and 19 patients were assigned to experimental arm B, with neoadjuvant FFX followed by surgery and adjuvant FFX. After inclusion of the 40 patients into the phase II part of the trial, the NEPAFOX trial was prematurely stopped owing to poor accrual.

**Fig. 1:**
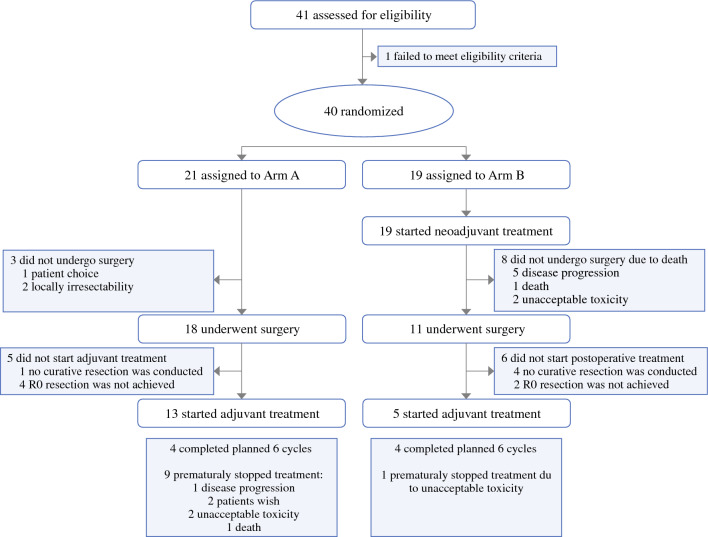
CONSORT diagram; all 40 patients who were randomly assigned were included in the intention-to-treat population, and the curative resected population comprised all patients who received radical oncological pancreatic surgery (*N* = 17 arm A, *N* = 7 arm B)

Baseline characteristics, including age, sex, and ECOG performance status, were similar between both arms. Before randomization, 76.2% of the patients in arm A and 78.9% in arm B were classified as primarily resectable, and the remaining patients were classified as borderline resectable. Jaundice was present at baseline in 33.3% (*n* = 7) of patients in arm A and 42.1% (*n* = 8) of patients in arm B. Of the seven patients with jaundice in adjuvant gemcitabine arm, four (57.1%) received presurgical stenting, and seven of the eight patients with jaundice in the perioperative FFX arm (87.5%) received biliary stenting (Fig. 1).

The median time from randomization until the start of systemic neoadjuvant therapy in arm B was 11 days (range 2–29 days), indicating a delay of tumor therapy that was most likely caused by interventional procedures to enable patients’ neoadjuvant approach, such as diagnostic histological tumor confirmation or biliary stenting. In arm A, 13 patients (62%) began the adjuvant systemic gemcitabine treatment (62%), with a median of three cycles applied. All 19 patients (100%) randomized to arm B started neoadjuvant FFX chemotherapy, with a median of 6.0 FFX cycles applied (pre- and postoperatively). Of 19 patients who started with FFX, 11 (57.9%) completed the recommended six preoperative cycles. Five patients (26%) started postoperative chemotherapy, with four completing all recommended cycles (21% of total population; Fig. 1) (Table 1).

**Table 1:** Baseline characteristics of the intention-to-treat population according to treatment group

	Arm A: adjuvant gemcitabine (*N* = 21)	Arm B: perioperative FOLFIRINOX (*N* = 19)
*Age*		
Mean ±SD	63.4 ± 9.79	63.3 ± 7.13
Median (range)	62.0 (46–84)	65.0 (49–76)
*Sex*		
Male	11 (52.4%)	12 (63.2%)
Female	10 (47.6%)	7 (36.8%)
*ECOG*		
ECOG 0	13 (61.9%)	12 (63.2%)
ECOG 1	8 (38.1%)	7 (26.8%)
*Resectability*		
Resectable	16 (76.2%)	15 (78.9%)
Borderline resectable	5 (23.8%)	4 (21.1%)
*Conditions before trial inclusion*		
Preoperative jaundice	7 (33.3%)	8 (42.1%)
—of these: biliary stent	4 (57.1%)	7 (87.5%)
*cT-stage*		
cTx	2 (9.5%)	1 (5.3%)
cT1	3 (14.3%)	3 (15.8%)
cT2	7 (33.3%)	7 (36.8%)
cT3	7 (33.3%)	5 (26.3%)
cT4	2 (9.5%)	3 (15.8%)
*N-stage*		
cNx	6 (28.6%)	2 (10.5%)
cN0	6 (28.6%)	7 (36.8%)
cN1	9 (42.9%)	10 (52.6%)
*UICC tumor stage*		
Ia	0	2 (10.5%)
IB	4 (19.0%)	3 (15.8%)
IIA	2 (9.5%)	1 (5.3%)
IIB	7 (33.3%)	8 (42.1%)
III	2 (9.5%)	3 (15.8%)
Unknown	6 (28.6%)	2 (10.5%)

*ECOG* Eastern Cooperative Oncology Group, *UICC* Union for International Cancer Control

Overall, 156 and 166 adverse events (AEs) were reported in arm A and arm B, respectively, and the incidence of AEs grade ≥ 3 was higher in arm B (57% versus 84%). However, only two AEs in arm A and one in arm B with grade ≥ 3 were classified as treatment related. The most common AEs with grades 1–2 (> 20%) were diarrhea and fatigue in both arms, fever in arm A, and anorexia, dizziness, and nausea in arm B. The most common AEs with grade ≥ 3 (< 5%) were neutropenia in arm A and abdominal pain, biliary tract infection, diarrhea, GGT increase, and sepsis in arm B (Table 2). One fatal serious adverse event (SAE) that was classified as related to the treatment was reported in arm A (hepatic failure) and two nonrelated fatal SAEs (both sepsis) occurred in arm B.

**Table 2:** Adverse events (related or not), maximum grade by patient and category

	Arm A: adjuvant gemcitabine (*N* = 21)			Arm B: perioperative FOLFIRINOX (*N* = 19)		
	Grades 1–2	Grades 3–4	Grade 5	Grades 1–2	Grades 3–4	Grade 5
Abdominal pain	2 (10%)	1 (5%)		1 (5%)	2 (11%)	
Anorexia	3 (14%)			4 (21%)		
Biliary tract infection					2 (11%)	
Diarrhea	5 (24%)	1 (5%)		3 (16%)	2 (11%)	
Dizziness	3 (14%)			4 (21%)		
Fatigue	5 (24%)	1 (5%)		5 (26%)		
Fever	5 (24%)	1 (5%)		3 (16%)		
GGT increased		1 (5%)		1 (5%)	2 (11%)	
Hepatic failure			1 (5%)			
Nausea	1 (5%)	2 (10%)		5 (37%)		
Neutropenia	1 (5)	3 (14%)		1 (5%)		
Sepsis					2 (11%)	2 (11%)

Except for grade 5, adverse events are displayed when they were observed in > 20% patients at grades 1 or 2 or > 5% patients at grades 3 or 4 in at least one of both arms

G-CSF was administered in the first cycle in the perioperative FFX arm in 12 patients (63.1%), with 4 (33%) experiencing a delay in the FFX schedule. Only one of these four patients had a delay in schedule owing to neutropenia (Table S1). Of the remaining seven patients who did not receive upfront G-CSF in cycle 1, three (37%) showed a dose delay, with one of them due to neutropenia. Of 19 patients in the perioperative FFX arm, 2 (10.5%) received iron substitution, each in cycle 2 of the neoadjuvant treatment (Table 3).

**Table 3:** Response measurements according to RECIST 1.1. in the neoadjuvant experimental arm B at the end of FFX neoadjuvant treatment

Response	Arm B: perioperative FOLFIRINOX (*N* = 19)
Complete response	0 (0%)
Partial response	3 (15.8%)
Stable disease	6 (31.6%)
Progressive disease	3 (15.8%)
Not done	7 (36.8 %)

According to RECIST 1.1, none of the patients in the perioperative FFX arm achieved complete response after completion of the neoadjuvant treatment. Three patients (15.8%) showed partial response, six patients (31.6%) showed stable disease, and three patients (15.8%) had signs of tumor progression. For seven patients (36.8%), response evaluation according to RECIST was not conducted, and three of these patients showed clinically progressive disease and did not proceed to surgery (Table 4). One patient with progressive disease following RECIST underwent surgery, but only liver metastases were resected. Overall, 18 patients (85.7%) in arm A and 11 patients (57.9%) in arm B proceeded to surgery. Reasons for not conducting surgery were patient’s wish and detection of local irresectability before surgery in arm A, as well as disease progression in arm B (Fig. 1). Of all patients undergoing surgery, 17 patients (94%) in arm A and 7 patients (64%) in arm B received radical tumor surgery, including pancreatic tumor resection and lymph node dissection. R0 resection was achieved in 5 of the 7 (76.5%) patients in arm A and in 13 of the 17 (71.4%) patients in arm B. Perioperative morbidity rate in all patients who underwent any kind of surgery was 72.2% in arm A and 45.5% in arm B. There were no relevant differences within the local and systemic complications between both arms (Table 4).

**Table 4:** Resection in patients who underwent surgical therapy including surgical margins in the intention-to-treat population according to treatment group

	Arm A: adjuvant gemcitabine (*N* = 21)	Arm B: perioperative FOLFIRINOX (*N* = 19)	Total (*N* = 40)
*Was surgery conducted*?			
No	3 (14.3%)	8 (42.1%)	11 (12.5%)
Yes	18 (85.7%)	11 (57.9%)	29 (72.5%)
If surgery	(*N* = 18)	(*N* = 11)	(*N* = 29)
*Was resection conducted?*			
No	1 (5.6%)	2 (18.7%)	3 (10.3%)
Yes	17 (94.4%)	9 (81.8%)	26 (89.7%)
*Was curative resection conducted?*			
Yes	17 (94.4%)	7 (63.6%)	24 (82.8%)
If curatively resected	(*N* = 17)	(*N* = 7)	(*N* = 24)
*R-status*			
R0-resection	13 (76.5%)	5 (71.4%)	18 (75.0%)
R1-resection	2 (11.8%)	0	2 (8.3%)
R2-resection	1 (5.9%)	0	1 (4.2%)
Rx	1 (5.9%)	2 (28.6%)	3 (12.5%)
*Surgical morbidity*	(*N* = 18)	(*N* = 11)	(*N* = 29)
Any local or systemic complications	13 (72.2%)	5 (45.5%)	18 (62.1%)
Secondary hemorrhage	0 (0%)	1 (9.1%)	1 (3.4%)
Anastomotic leak	1 (5.6%)	1 (9.1%)	2 (6.9%)
Fistula	2 (11.1%)	0 (0%)	2 (6.9%)
Abscess	1 (5.6%)	0 (0%)	1 (3.4%)
Other local complications^a^	3(16.7%)	2 (18.2)	5 (17.2%)
Pneumonia	1 (5.6%)	1 (9.1%)	2 (6.9%)
Sepsis	0 (0%)	2 (18.2%)	2 (6.9%)
Other systemic complications^b^	2 (11.1%)	0 (0%)	2 (6.9%)
Surgical mortality			
24-day mortality	0 (0%)	1 (9.1%)	1 (3.4%)
64-day mortality	0 (0%)	1 (9.1%)	1 (3.4%)

^a^Other local complications for arm A include: tumor-related cholestasis with exocrine pancreatic insufficiency; for arm B: portal vein thrombosis with refractory ascites and protein deficiency

^b^Other systemic complications for arm A include: hypokalemia, thoracic aortic aneurysms, and peripheral edema with diuresis and fever up to 39 °C

**Fig. 2:**
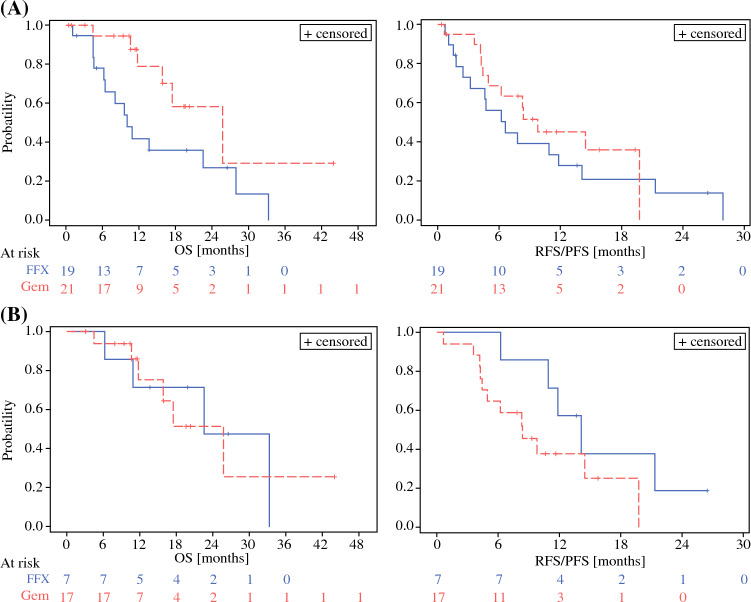
Overall and recurrence/progression-free survival; overall survival and recurrence/progression-free survival in the intention-to-treat (ITT) (**A**) and the curative resected population (**B**) in the adjuvant gemcitabine (Gem) arm A versus the FOLFIRINOX (FFX) arm B

Within the ITT population, the median OS was increased in arm A compared with arm B [25.68 months (95% CI 11.74, –) versus 10.03 months (95% CI 6.25–27.95)]. The median RFS/PFS was 9.8 months (95% CI 4.44–19.76) in arm A and 6.64 months (95% CI 2.50–11.84) in arm B (HR 0.722; Fig. 2). However, within the population of patients who received curative surgery, median OS was comparable between both arms [arm A: 25.68 months (95% CI 11.74, –); arm B: 22.55 months (95% CI 6.25, –); HR 1.054]. Furthermore, median RFS/PFS was numerically increased in the patients receiving neoadjuvant FFX [arm A: 8.38 months (95% CI 4.27, –), arm B: 14.14 months (95% CI 6.25, –; Fig. 2)].

## Discussion

Robust data based on phase III RCTs established the adjuvant therapy approach for pancreatic cancer treatment. These data have been shown to improve survival, but only for patients who are fit enough after surgery. Unfortunately, a subset of patients who undergo upfront surgery do not recover adequately to be able to receive adjuvant treatment. Thus, about 45% of patients scheduled for adjuvant chemotherapy after surgical resection do not receive it owing to poor performance status, postoperative morbidity, or early disease progression.^18,19^ In addition, the majority of data regarding perioperative tumor therapy show a definite survival benefit of chemotherapy in both the neoadjuvant and adjuvant settings compared with data of no perioperative chemotherapy.

The results of NEPAFOX show a shorter median survival in the perioperative FFX arm compared with the adjuvant gemcitabine standard arm in the intention-to-treat population, presumably because fewer patients received surgery for a variety of reasons. Thus, within the limits of this defined trial population, FFX neoadjuvant treatment was not shown to provide longer survival than upfront surgery followed by adjuvant gemcitabine. However, almost all patients in the standard arm A without neoadjuvant treatment, but only one-third of the patients who received neoadjuvant FFX treatment in arm B, underwent curative surgery. The main reason for not conducting surgery for patients in arm B was progression under neoadjuvant treatment. Among these patients who received curative resection, the use of perioperative FFX compared with adjuvant gemcitabine alone resulted in an almost doubling of RFS/PFS (14.1 months vs 8.4 months) and no difference in OS. The results are in line with previous data showing median disease free-survival ranging between 10 months and 17 months for perioperative chemotherapy in resectable PDA.^20–22^ Thus, the results of the NEPAFOX underlines patient selection for neoadjuvant therapy.

Several trials have been performed to assess neoadjuvant chemotherapy for pancreatic resectable disease. However, most of the early trials were only cohort trials and used older single-agent chemotherapy regimens, plus or minus radiation.^23–33^ Recent pooled data from three randomized controlled phase II trials, however, suggested that neoadjuvant therapy for resectable pancreatic cancer could be the new standard of care^15^

During the conduct of the NEPAFOX trial, the results of the PRODIGE 24/CCTG PA.6 phase III trial were published, demonstrating that the modified FFX significantly increased the efficacy in the adjuvant setting compared with gemcitabine monotherapy. Furthermore, the mFFX regimen was superior to gemcitabine monotherapy in all subgroups analyzed, including patients with advanced T3/4 tumors, N+ tumors, and even after R1 resection.^34^ In addition, the gemcitabine only treatment achieved a 10–15-month longer overall survival compared with previous trials evaluating adjuvant gemcitabine, which was likely owing to the high use of FFX after relapse in the gemcitabine arm.^34^ The PRODIGE 24/CCTG PA.6 trial was therefore the first, and so far the only, study providing such a benefit in a group with positive surgical margins. However, it must be noted that the median age of 63 years in this trial suggests that the majority of patients included were younger than the average population of patients with PDAC, e.g., 69 years in 600 patients of the QOLIXANE trial.^35^

In addition, despite the promising results for adjuvant treatment, it is important to note that only about 60% of patients with PDAC receive adjuvant treatment in real-world settings owing to perioperative morbidity, even when the start of adjuvant treatment is delayed up to 3 months postoperatively.^36^ Therefore, trial populations of adjuvant studies are highly selected patients.

One-quarter of the NEPAFOX population was defined as borderline resectable, and while adjuvant chemotherapy remains the standard approach for primarily resectable pancreatic cancers, borderline resectable or even locally advanced pancreatic cancers are increasingly treated with neoadjuvant chemotherapy.^37,38^ The intention of neoadjuvant chemotherapy in borderline resectable pancreatic cancer is, on the one hand, to decrease tumor size for an easier resection of the primary tumor and to achieve an R0 resection, and on the other hand, to biologically select patients without tumor progression under upfront systemic therapy who will benefit from subsequent radical tumor surgery. Previous trials have even shown favorable outcomes after a neoadjuvant approach followed by surgery, although post-neoadjuvant imaging suggested a persistent unresectability. This indicates that surgical evaluation will always be an option when surgical intervention is the only possibility to cure a patient and achieve long-term survival.^37,39^

Despite the promising increase in RFS/PFS in the curative resected population under perioperative FFX treatment, the use of FFX in the neoadjuvant situation is not entirely unproblematic. If the neoadjuvant concept fails, a potent therapy for the then palliative situation has already been exhausted. In addition, one must also be aware that in resectable pancreatic cancers, neoadjuvant therapy could lead to a delay of the required surgery, especially when patients suffer complications from the neoadjuvant systemic therapy. As such, cholestasis could lead to the need of biliary stenting, enabling patients to receive a cytotoxic therapy and provoke a further delay of surgery. According to the current guidelines, surgery of pancreatic cancer can be performed without prior histological confirmation.^40,41^ However, histological confirmation of the primary tumor with a need of a positive biopsy is required before the start of a systemic therapy, especially neoadjuvant systemic chemotherapy. This often turns out to be not so succinct, because unlike, for example, the relatively simple biopsy of liver metastasis in stage IV disease, biopsy of primary pancreatic metastasis is always required in clear curative situations.^42^ In up to 25% of cases there is no sufficient material obtainable even after repeated endoscopic-ultrasound-guided fine needle aspiration, and therefore surgery gets delayed without even the option of the planned neoadjuvant chemotherapy treatment being initiated.^43^ Furthermore, the histological structure of the primary pancreatic cancer with dense stromal character may limit the effectiveness of upfront chemotherapy on the primary cancer.^44^ Thus, patient selection is critical for neoadjuvant treatment to reduce the risk of neoadjuvant concept fails that would limit remaining treatment options.

Thus, the rationale of a potential benefit owing to early systemic treatment in the form of a neoadjuvant treatment should be weighed against the complications and toxicity of the treatment potentially affecting perioperative morbidity and even mortality. In our trial, no increase in toxicity regarding surgical complications in the neoadjuvant treatment arm compared with the standard arm were observed.

### Supplementary Information

Below is the link to the electronic supplementary material.

Supplementary file1 (DOCX 14 kb)

## Data Availability

The data underlying this article will be available from the corresponding author upon reasonable request.
