# Supplementary material for: Adjuvant Gemcitabine Versus Neoadjuvant/Adjuvant FOLFIRINOX in Resectable Pancreatic Cancer: The Randomized Multicenter Phase II NEPAFOX Trial
Source: Ann Surg Oncol. 2024 Mar 8;31(6):4073–83. doi: 10.1245/s10434-024-15011-7 (PMC11076394; doi:10.1245/s10434-024-15011-7)
Supplement: Supplementary file 1 — Supplementary file1 (DOCX 14 kb) [file 10434_2024_15011_MOESM1_ESM.docx]

**Supplementary**

**Table S1: Number of FFX-treated patients in arm B who received G-CSF**

|  | Cycle | Number of patients treated with FFX | Number (percent) of patients received G-CSF |
| --- | --- | --- | --- |
| Neoadjuvant | Cycle 1 | 19 | 12 (63%) |
|  | Cycle 2 | 16 | 11 (69%) |
|  | Cycle 3 | 15 | 15 (100%) |
|  | Cycle 4 | 15 | 12 (80%) |
|  | Cycle 5 | 12 | 9 (75%) |
|  | Cycle 6 | 11 | 8 (73%) |
| Adjuvant | Cycle 1 | 5 | 3 (60%) |
|  | Cycle 2 | 5 | 3 (60%) |
|  | Cycle 3 | 5 | 3 (60%) |
|  | Cycle 4 | 4 | 2 (50%) |
|  | Cycle 5 | 4 | 2 (50%) |
|  | Cycle 6 | 4 | 2 (50%) |
